# Lawrence Bogorad's scientific contributions and remembrances on his 100^th^ birth anniversary

**DOI:** 10.1111/pbi.13719

**Published:** 2021-11-23

**Authors:** Kailash C. Bansal

**Affiliations:** ^1^ National Academy of Agricultural Sciences New Delhi India

**Keywords:** Lawrence Bogorad, chloroplasts, chloroplast DNA, molecular biology, chloroplast genes

## Abstract

Lawrence Bogorad, much known for his fundamental discoveries in plant molecular biology, reached the pinnacle of research as a topmost international scientist, occupying several key scientific leadership positions in his five‐decade‐long association with plant science community. Much has been said and written about his life and his dedication to science as a pioneer in photosynthesis research. Here, we reminisce some of his key groundbreaking scientific contributions, and share our experiences and appreciation for his mentorship through personal messages, as we celebrate his 100^th^ birth anniversary that falls on 29 August 2021.

## Introduction and early research

**Figure 1:**
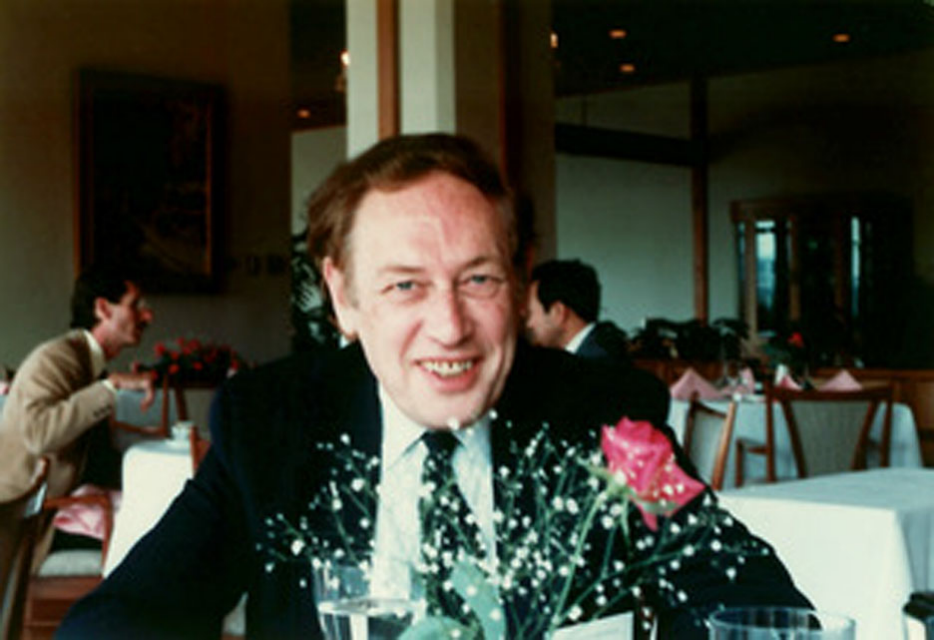
Lawrence (Laurie to his friends and family) Bogorad at the Oji International Seminar ‘New Aspects of Plant Cell Biology and Molecular Biology’, held in October 1986, at a resort Iseshima in Japan. (Source: Hiro Kobayashi, Japan).

**Figure 2:**
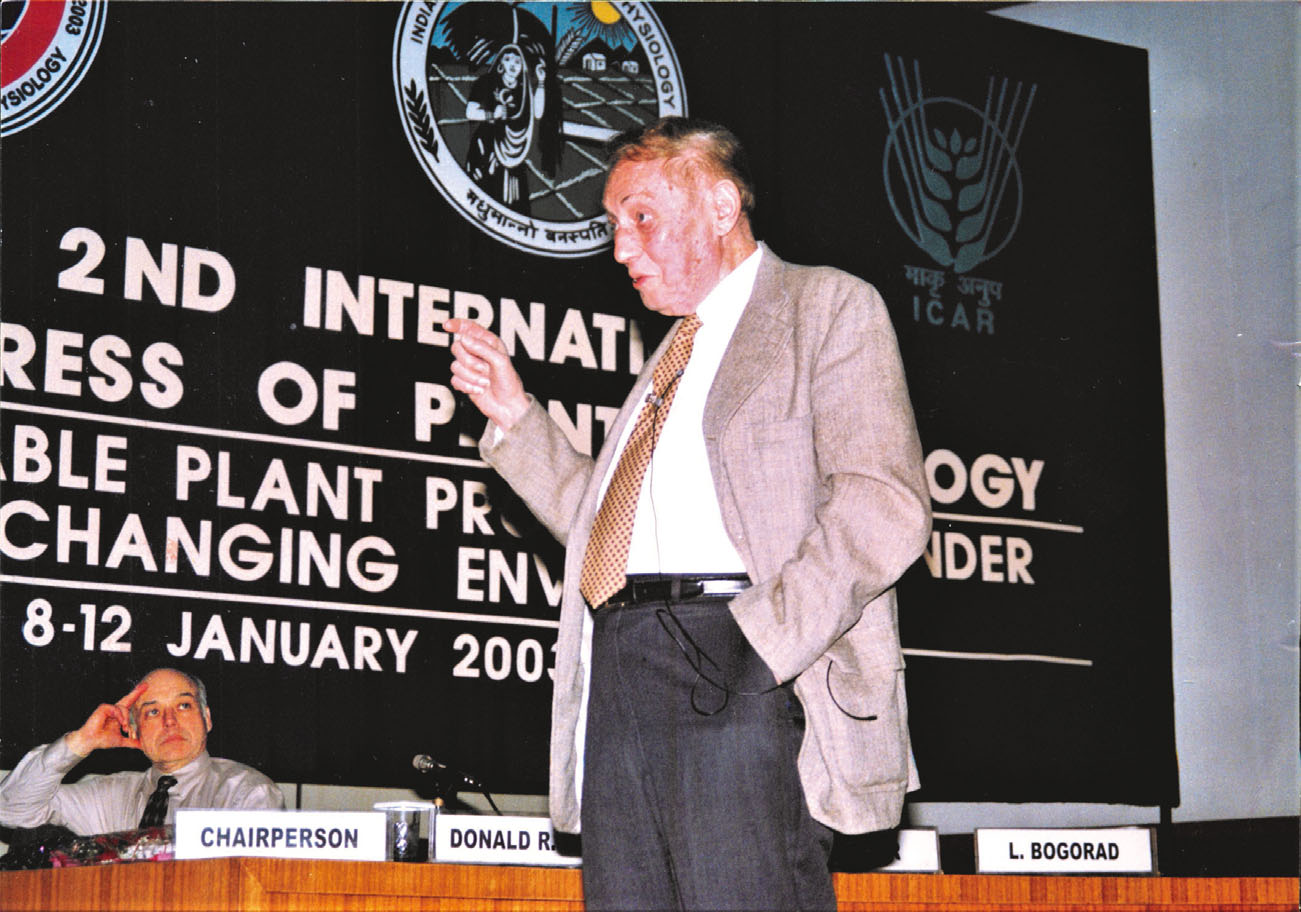
Professor Lawrence Bogorad delivering a plenary talk at the second International Congress of Plant Physiology in New Delhi, India, in January 2003 (Chairperson: Dr. Donald R. Ort is also seen in the picture) (Source: Madan Pal, Indian Society for Plant Physiology, IARI, New Delhi, India).

Lawrence Bogorad was an internationally acclaimed scientist (Figure 1). His career in science spanned over five decades, 1953–2003 (Table 1, Figure 2). He made path‐breaking discoveries in the area of plant molecular biology with a special focus on chloroplast biogenesis and understanding the basics of photosynthetic apparatus in plants and algae (Bogorad, 2001a, 2003; Rodermel *et al*., 2005; Swift, 1985), with a distinct goal of application in agricultural biotechnology (Bogorad, 2000; also see Bogorad, 2005).

**Table 1:** Lawrence Bogorad's 50 years of glorious scientific career (1953–2003)

Year	Position	Area of research
1951–1953	Fellow, Rockefeller Institute for Medical Research (now Rockefeller University, New York, NY), USA (Sam Granick's Lab)	Biosynthesis of porphyrins in algal cells
1953	Assistant Professor, Department of Botany, University of Chicago, Chicago, IL, USA	Tetrapyrrole biosynthesis
1960	Fulbright Scholar, CSIRO (Commonwealth Scientific and Industrial Organization), Canberra, Australia	–
1961	US National Science Foundation (NSF) Senior Postdoctoral Fellow at the Karolinska Institute in Stockholm, Sweden	–
1961	Professor, Department of Botany, University of Chicago, Chicago, IL, USA	Biosynthesis of porphyrins
1967	Professor, Biology, Harvard University, Cambridge, MA, USA	Genetics of Chlamydomonas chloroplast ribosomes
1974–1976	Chairman of the Department of Biological Sciences at Harvard University, Cambridge, MA, USA	Mapping ribosomal DNAs on maize chloroplast chromosome
1976	Director of the Maria Moors Cabot Foundation, Harvard, University, Cambridge, MA, USA	Expression analysis of the entire chloroplast chromosomal regions and specific genes in relation to illumination of dark‐grown maize seedlings
1980–1991	Maria Moors Cabot Professor of Biology, Harvard, University, Cambridge, MA, USA	Maize chloroplast RNA polymerase using DNA cloning technologies
1991–2003	Maria Moors Cabot Professor of Biology Emeritus, Harvard, University, Cambridge, MA, USA	Transcriptional regulation of photosynthetic genes

During his postdoctoral training (1951–1953), Bogorad worked on biochemical characterization of the porphyrin biosynthesis pathway, which he continued after joining as an Assistant Professor in the University of Chicago. He made pioneering contributions towards porphyrin biosynthesis, which are well acclaimed. The first unequivocal evidence of enzymatic conversion of porphobilinogen to uroporphyrinogen came from his laboratory (Bogorad, 1958a, 1960), and also that two enzymes participate in the synthesis of uroporphyrinogen III from porphobilinogen. Bogorad (1958a) isolated and purified the first enzyme, uroporphyrinogen I‐synthetase from spinach leaf tissue, with catalytic role of converting porphobilinogen into uroporphyrinogen I. The second enzyme isolated from aqueous extracts of wheat germ was identified as uroporphyrinogen III‐cosynthetase (Bogorad, 1958b). Furthermore, it was shown that uroporphyrinogen I was not a substrate for the uroporphyrinogen III‐cosynthetase and the action of both the enzymes was needed to convert porphobilinogen to uroporphyrinogen III (Bogorad and Marks, 1960a), which was later confirmed by others working with *Rhodopseudomonas spheroides* extracts (Jordan and Shemin, 1973).

These studies by Bogorad also shed light on the mechanism of enzymatic production of uroporphyrinogen I and III from porphobilinogen, ruling out the possibility of the involvement of formaldehyde as a reactant or a product (Bogorad and Marks, 1960b). Furthermore, Bogorad's investigations clearly showed that opsopyrrole‐dicarboxylic acid and isoporphobilinogen have no role in the enzymatic formation of uroporphyrinogen I or III (Bogorad, 1960, 1960b), as against earlier assumption by several workers that these two pyrroles function as coenzymes of uroporphyrinogen III cosynthetase (Carpenter and Scott, 1959).

**Figure 3:**
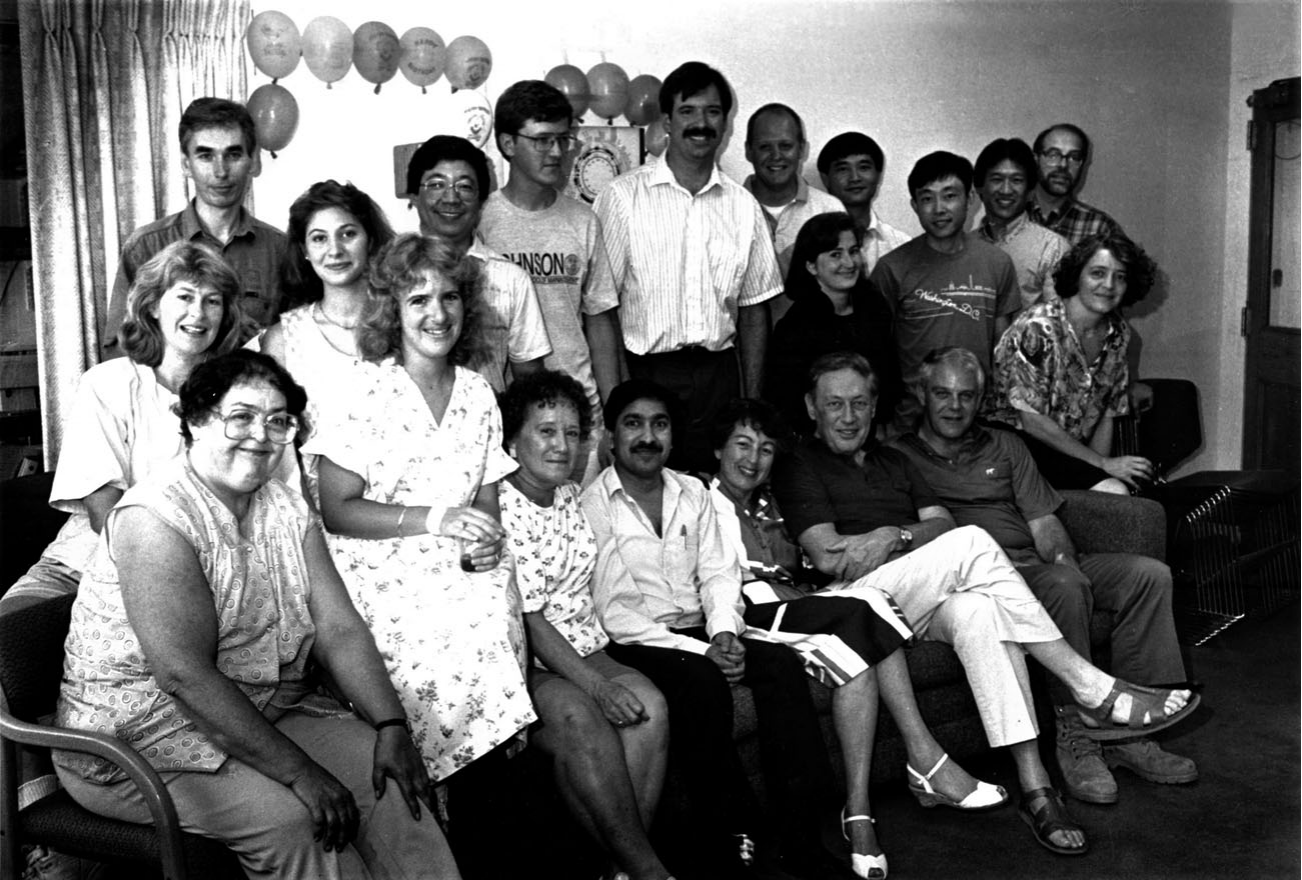
Lab members gathered for celebrating Lawrence Bogorad's 70^th^ birthday on 29 August, 1991, Harvard Biolabs, Cambridge, MA, USA. First row (from the left to the right): Claire, Gisèle Rubín, Olga Milli, Kailash Bansal, Rosalyn Bogorad, Lawrence Bogorad, Robert Troxler, Marisa Salvador. Second row (from the left to the right): Jean Haley, Uwe Klein, Elisabeth Battinelli, Je Chang Woo, Jean‐Frederic Viret, Thomas Templeman, James DeCamp, Dolores Maria Abarca, Xing Su, Sheng Luan, Don Jin, Arthur (Source: Julie O'Neil, Dr. Bogorad's secretary).

**Figure 4:**
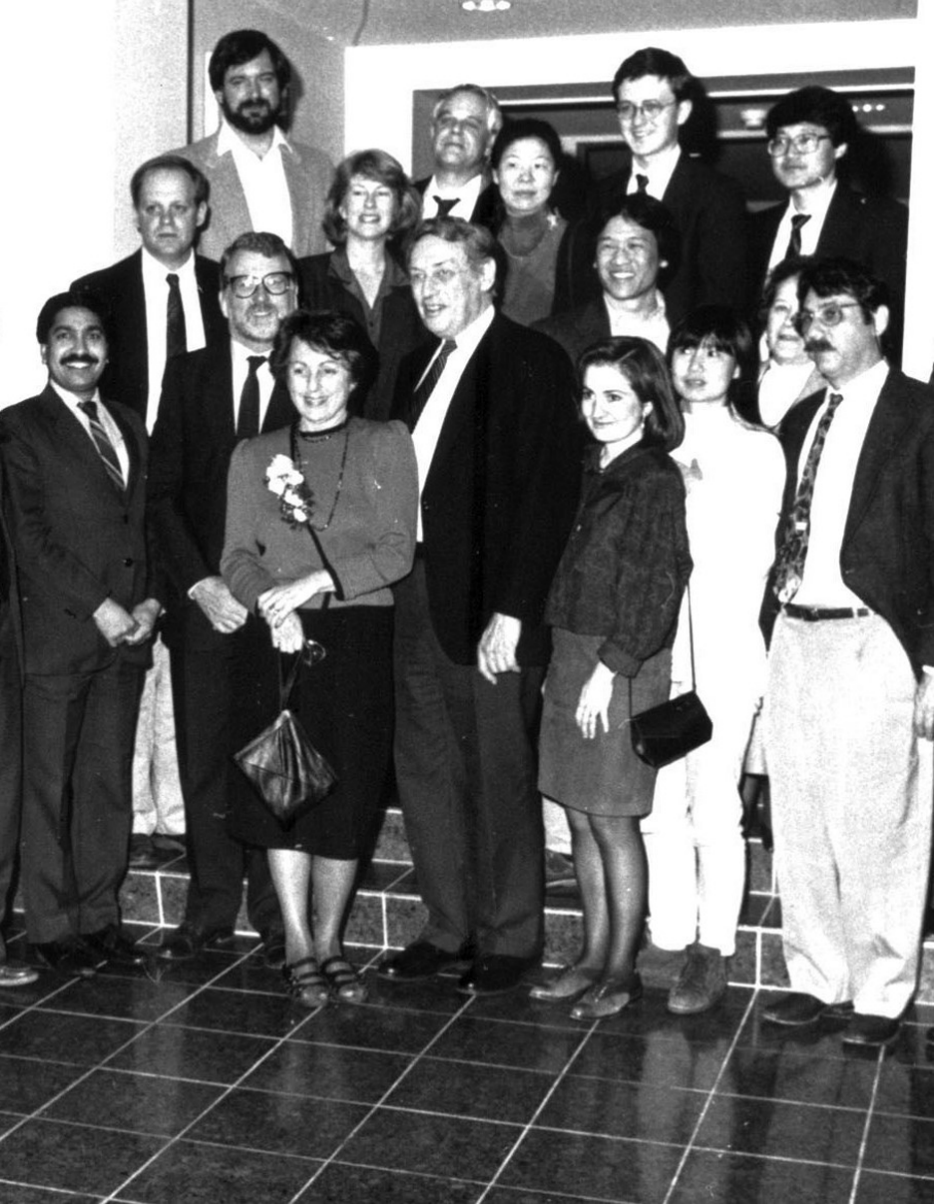
Professor Lawrence Bogorad and his Lab members with Dr. Jeff Schell (University of Gent, Belgium) at Harvard Biolabs, Cambridge, MA, USA, 1992. Dr. Jeff Schell was invited to deliver a lecture to mark Bogorad's retirement. First row (from the left to the right): Kailash Bansal, Jeff Schell, Rosalyn Bogorad, Lawrence Bogorad, Dolores Maria Abarca, Jen Sheen, Fred Ausubel. Second row (from the left to right): James DeCamp, Jean Haley, Alice Cheung, Don Jin, Olga Milli. Third row (from the left to the right): Thomas Templeman, Robert Troxler, Jean‐Frederic Viret, Dong‐Hee Lee (Source: Julie O'Neil, Dr. Bogorad's secretary).

**Figure 5:**
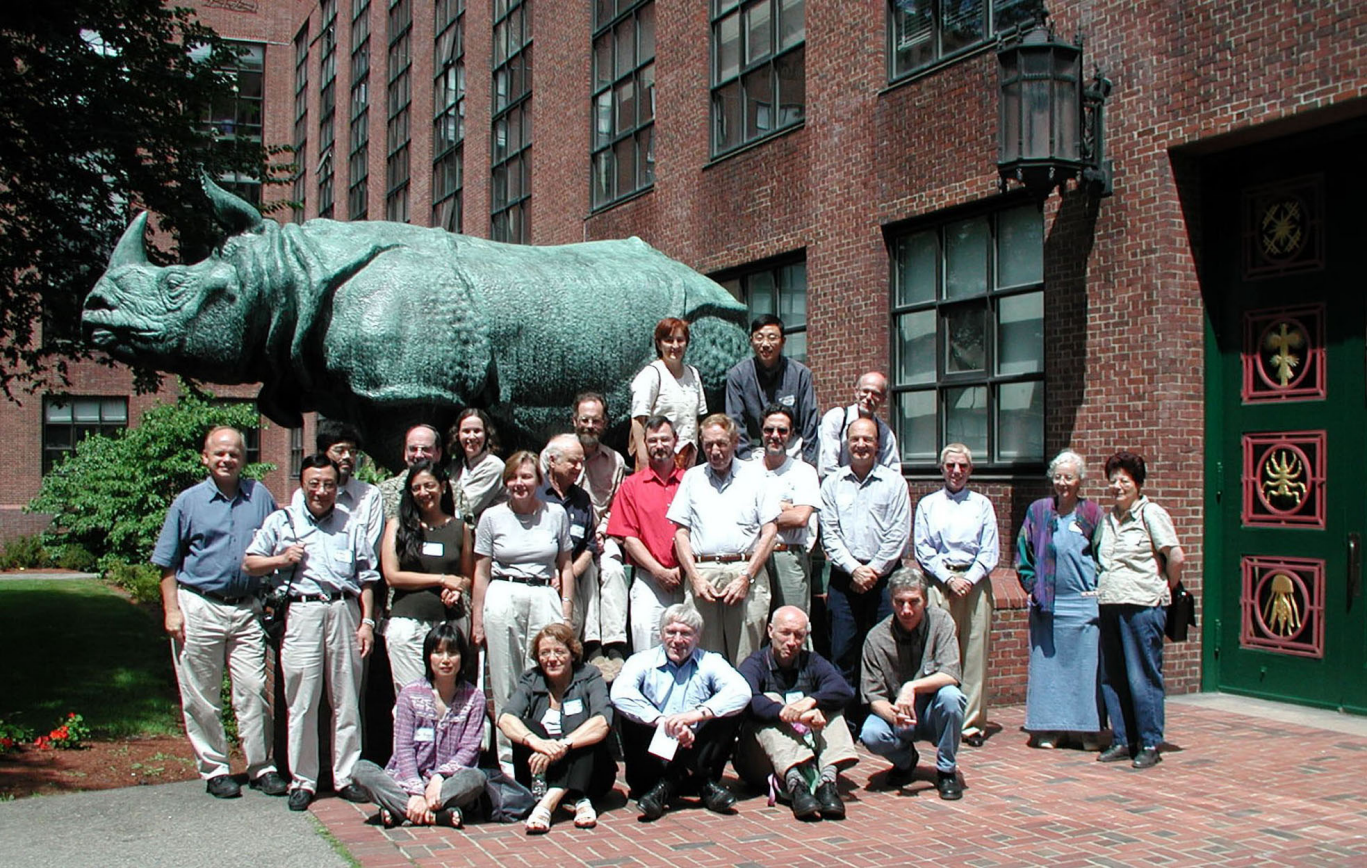
A group photograph at the Fourth Lawrence Bogorad Symposium coordinated by Andre Steinmetz and held in July 2001 in Cambridge, MA, USA. First row sitting (from the left to the right): Jen Sheen, Marisa Salvador, Andre Steinmetz, Apel Klaus and Uwe Klein. Second row (from the left to the right): Sabeeha Merchant, Joanne Barton, Neil Schultes, Lawrence Bogorad and John Bedbrook. Third row (from the left to the right): Richard (Dick) Sayre, Hiro Kobayashi, Harry Roy, Karen Muskavitch, Carl Price, Enno Krebbers and Iggy Larrinua. Fourth row (from the left to the right): Dolores Maria Abarca, Sheng Luan and Gerhard Link (Source: Hiro Kobayashi, Japan).

After the discovery of chloroplast DNA in the 1960s, Bogorad's research focus shifted towards understanding the role of chloroplast DNA, ribosomes and RNA synthesis for unravelling the details of plastid biology and in visualizing the link between the modern‐day chloroplasts with their cyanobacterium‐related endosymbiont ancestors. In 1967, Bogorad moved to Harvard University, and established a vibrant top‐ranking research group for studying chloroplast biology using the tools of molecular biology (Table 1, Figures 3, 4, 5; also see Bogorad, 2005).

## Birth of molecular biology of chloroplasts

Some of his early observations are related to plastid biology, such as: (i) chloroplasts divide in situ and resemble cyanobacteria (blue–green algae); (ii) some features of chloroplasts are transmitted maternally in a non‐Mendelian manner; (iii) chloroplasts contain their own genetic information; (iv) chloroplasts might have arisen as endosymbionts derived from blue–green alga‐like organisms; and (v) that chloroplasts are the sites of photosynthetic oxygen production and chlorophyll is the photoreceptor in leaves and green algae. A list of his lab's pioneering contributions in the area of plastid molecular biology, including his early research accomplishments, is given in Table 2.

**Table 2:** Discovery milestones from Dr. Bogorad's laboratory spanning over 50 years

Year	Discovery milestone	References
1952	Isolated a Chlorella mutant for revealing precursors of protoporphyrin	Bogorad and Granick (1952)
1953	Two enzymes involved in haem and chlorophyll biosynthesis, that is, porphobilinogen (PBG) deaminase and Uroporphyrinogen (Uro) III cosynthase	Bogorad and Granick (1953)
1960	Isolated variously pigmented mutants of *Cyanidium caldarium* (a red alga) useful for analysing the synthesis of phycocyanin and light induction of chlorophyll	Nichols and Bogorad (1960)
1963	Occurrence of RNA in plastids from etiolated and green maize leaves Localization of ribosomes in stroma of chloroplasts of maize	Jacobson *et al*. (1963)
1971	Genes encoding plastid ribosomal components are distributed between the organelle and the nucleus, indicating that plastids are semi‐autonomous organelles	Mets and Bogorad (1971) Mets and Bogorad (1972) Hanson and Bogorad (1977a,b); Hanson and Bogorad (1978)
1975	Suggested that endosymbiosis involved the transfer of genes from the symbiont to the host and/or that functions of symbiont genes came to be substituted for by nuclear genes	Bogorad (1975)
1976	Constructed the first restriction map of maize plastid DNA Multiple circles of chloroplast chromosome in a single chloroplast are genetically identical	Bedbrook and Bogorad (1976)
1976	Increase in chloroplast RNA polymerase activity during the greening etiolated maize seedlings	Apel and Bogorad (1976)
1977	Mapped genes for chloroplast rRNAs—16S and 23S Mapped and cloned the rbcL plastid gene encoding the plastid protein LS of Rubisco	Bedbrook *et al*. (1977) Coen *et al*. (1977)
1977	Maize chloroplast ribosomal RNA genes are part of a 22 000 base pair inverted repeat	Bedbrook *et al*. (1977)
1978	Mapped a gene on plastome, termed as ‘PG32’ (photoregulated) encoding a 32‐kD protein product (now called psbA)	Bedbrook *et al*. (1978)
1978	Tissue‐specific control of the transcription of rbcL in the chloroplasts of bundle sheath and mesophyll cells of mature maize leaves	Link *et al*. (1978)
1980	The first plant gene sequenced—the rbcL plastid gene encoding the plastid protein LS of Rubisco (a product of nuclear‐chloroplast chromosome collaboration)	McIntosh *et al*. (1980)
1982	Cloned and sequenced maize atpBE gene encoding beta and epsilon subunits of the chloroplast ATP synthase and that both subunits are fused	Krebbers *et al*. (1982)
1982	Mapped genes for rRNAs and tRNAs on the plastid genome and subsequently characterized	Steinmetz *et al*. (1982); Subramanian *et al*. (1983)
1985	Regions of the plastid genome containing photogenes identified and showed transcriptional control of different photogenes expression in maize Transcriptional control of the nuclear gene (*rbc*S) lags behind that of the chloroplast (*rbc*L) and may be regulated by plastid metabolism	Rodermel and Bogorad (1985)
1985	Discovered two homologous adjacent genes (now psaA and psaB) encoding two homologous proteins of the core of maize PS I reaction centre	Fish *et al*. (1985) Fish and Bogorad (1986)
1985	Systematic cloning, identification and molecular regulation of nuclear photosynthetic genes in the dimorphic bundle sheath and mesophyll cells of C4 maize leaves	Sheen and Bogorad (1985, 1986a, 1986b, 1987a, 1987b, 1988) Sheen *et al*. (1987)
1986	Metal ion regulation of the expression of photosynthetic proteins	Merchant and Bogorad (1986a, 1986b)
1988	Regulation of Large Subunit (of Rubisco) abundance by Small Subunit protein abundance in plastids at the level of rbcL mRNA translation	Rodermel *et al*. (1988) Rodermel (1999)
1988	Converted a chloroplast gene into a functional nuclear gene	Cheung *et al*. (1988)
1990s	Maize chloroplast rpo genes encode functional components of the chloroplast RNA polymerase enzyme Sigma factors as components of chloroplast RNA Polymerase enzyme	Hu and Bogorad (1990) Hu *et al*. (1991) Troxler *et al*. (1994)
1992	Identified cis‐sequences regulating light‐, developmental‐, tissue‐ and cell‐specific transcription of photosynthetic genes in maize leaves	Luan and Bogorad (1992); Bansal *et al*. (1992); Bansal and Bogorad (1993); Viret *et al*. (1994); Purcell *et al*. (1995); Xu *et al*. (2001)
1990–2001	Identified cis‐sequences controlling plastid transcription and transcript stability in Chlamydomonas	Blowers *et al*. (1990), Blowers *et al*. (1993); Klein *et al*. (1992), Klein *et al*. (1994); Salvador *et al*. (1993); Singh *et al*. (2001)

A new genetic approach to studying chloroplast genes was brought into the lab by Bogorad's graduate students, Laurens Mets, Jeffrey Davidson and Maureen Hanson, who began mapping the coding location of chloroplast ribosomal proteins by selecting for antibiotic‐resistant mutants of *Chlamydomonas reinhardtii*. This project resulted in the important finding that some components of the chloroplast ribosomes are encoded in chloroplast genes, whereas others are in the nuclear genes (Hanson and Bogorad, 1977a, 1977b; Mets and Bogorad, 1971, 1972). Specific experiments by Davidson *et al*. (1974) provided strong evidence that the nuclear ery‐M1 (erythromycin‐resistant mutant) gene encoded a chloroplast ribosomal protein that made the ribosome resistant to erythromycin. This seminal paper described for the first time that a chloroplast ribosomal protein is encoded by a nuclear gene and not by a chloroplast gene. This raised questions about the independence of contemporary chloroplasts, and prompted Bogorad (1975, 1982) to conclude ‘that if plastids did indeed arise as endosymbionts, some of their genes had been transferred to the nucleus and/or some nuclear‐encoded cytoplasmic proteins that originated in the host came to substitute for organelle or chloroplast proteins’.

It was proved unequivocally, already in the 1960s, that chloroplasts contained their own DNA and are able to perform *in organello* protein synthesis (see the milestone Table on the chloroplast genome & genetic engineering, Daniell *et al*., 2021). After the discovery of restriction endonucleases, followed by the Southern hybridization procedure in the early 1970s, it became possible to map DNA. After John Bedbrook had joined Bogorad's lab, as postdoc, the first map of maize chloroplast DNA as a circular molecule was made, using the recognition sites for the restriction endonucleases *Sal*l, *Bam*HI and *Eco*RI (Bedbrook and Bogorad, 1976).

Soon thereafter, the structural features of the chloroplast DNA showed unequivocally that there was one kind of circle in maize chloroplasts with a 22‐kbp inverted repeat sequence, which contained genes for rRNAs. The rRNA genes were the first genes to be mapped physically on a chloroplast chromosome (Bedbrook *et al*., 1977). During the next few years, mapping, identification, cloning and sequencing of chloroplast genes permitted the use of these genes as templates for RNA polymerase research. In the early 1980s, the expression of the regions of the entire maize chloroplast chromosome and of specific genes in relation to illumination of dark‐grown seedlings was accomplished (Rodermel and Bogorad, 1985).

We note that Bogorad's group mapped the first protein encoding gene *rbc*L (encoding the large subunit of Rubisco, LSU) on the chloroplast chromosome (Bedbrook *et al*., 1979; Coen *et al*., 1977). In quick succession, the second chloroplast protein gene was mapped, which was a photoregulated gene (now designated as psbA; Bedbrook *et al*., 1978). The discovery of psbA gene (PG32) encoding the 32 kD protein, which turned out to be the D1 reaction centre protein of Photosystem II (PSII), gave birth to the field of light‐regulated chloroplast biogenesis and light‐induced expression of photosynthetic genes (Bedbrook *et al*., 1978; Fish *et al*., 1985). Several photogenes were later cloned and characterized after the complete chloroplast genome sequence had become available in the mid‐1980s.

The rbcL gene was the first plant protein gene to be sequenced in Bogorad's laboratory (McIntosh *et al*., 1980). Several other chloroplast genes encoding photosynthetic proteins were later identified based on the use of antibodies and Western blotting experiments (Fish *et al*., 1985; Haley and Bogorad, 1989). Another significant contribution from Bogorad's lab was a metal ion regulation of the expression of photosynthetic proteins. Merchant and Bogorad (1986a,b) showed copper‐mediated regulation of the accumulation of two interchangeable electron carriers, plastocyanin and cytochrome c552 (cytochrome c6) in *Chlamydomonas reinhardtii*. It was further revealed using a *C*. *reinhardtii mutant* lacking plastocyanin that the expression of cytochrome c‐552 is directly controlled by Cu(II) concentration in the chloroplasts (Merchant and Bogorad, 1987).

**Figure 6:**
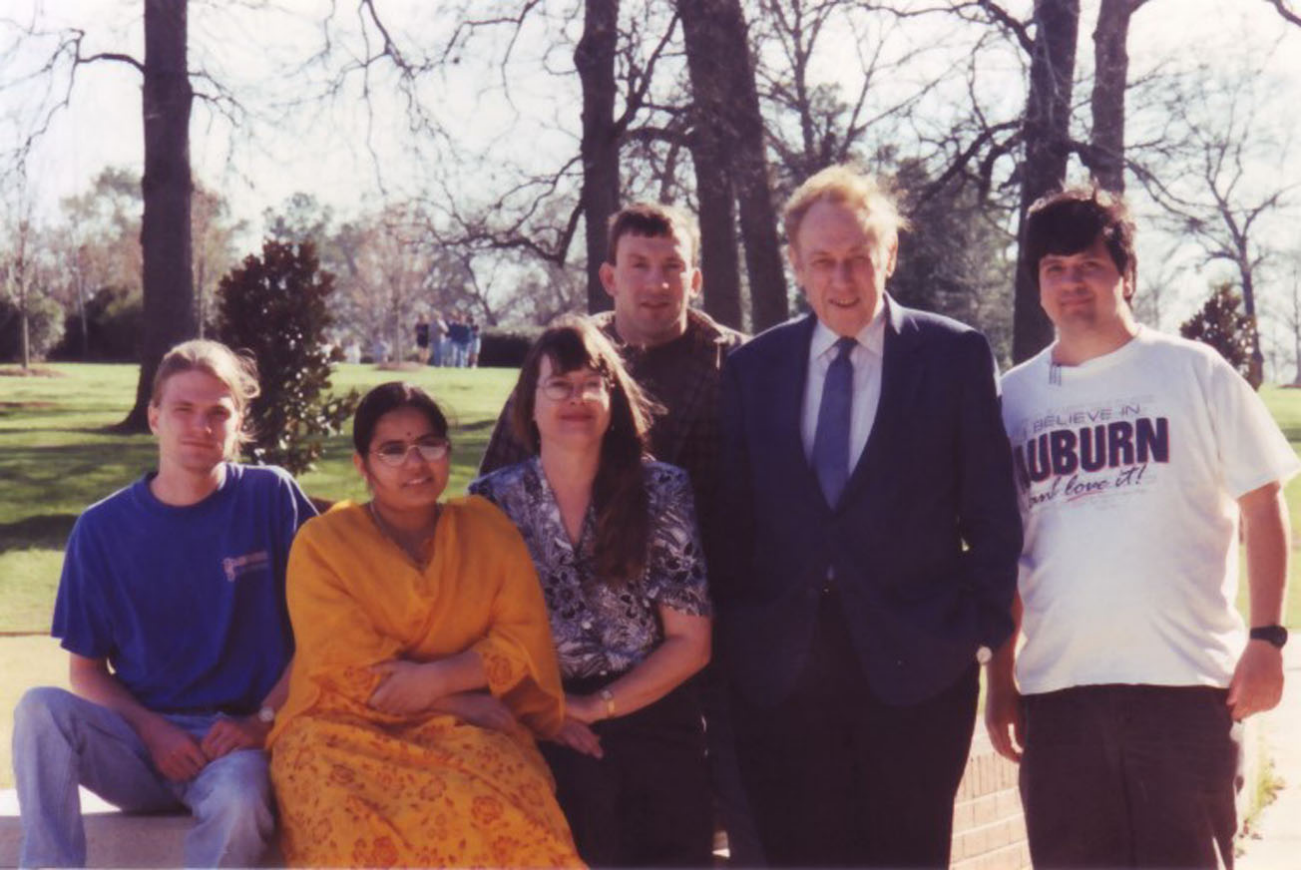
Lawrence Bogorad visiting Henry Daniell lab at Auburn University, Alabama, USA, 1995 (from the left to the right): Steven Gray, Madhuri Kota, Janie Brixey, Tommy Abner, Lawrence Bogorad and Sam Verma (Source: Henry Daniell, Pennsylvania, PA, USA).

Soon thereafter, Bogorad was quite impressed with the rapid advances in chloroplast molecular biology field; in the introduction of his book on ‘Molecular Biology of Plastids (1991), he optimistically stated that ‘the rapid acquisition of knowledge about unexplored aspects of plastid biology will accelerate as a result of recent advances in genetically transforming and manipulating the chloroplast genome’. Indeed, his pioneering work in transforming *Chlamydomonas* chloroplast genome (Blowers *et al*., 1989, 1990; Boynton *et al*., 1988) led to transformation of plant chloroplast genome, and the expression of high value proteins and enzymes that have been currently launched as commercial products or advanced to the clinic level (Daniell *et al*., 2021; see Figure 6).

By the late 1980s, Bogorad's group had developed a keen interest in identifying and characterizing nuclear genes encoding chloroplast proteins involved in the process of oxygenic photosynthesis, for example, *psa*A, *psa*B, *rbc*S, and his research team moved on to identify the *cis*‐acting elements controlling the expression of both chloroplast and nuclear genes using the transgenic approach.

## Functional characterization of photosynthetic genes

The availability of methods of genetic transformation during the 1980–1990 period for work in model photosynthetic organisms such as cyanobacteria, green alga *Chlamydomonas reinhardtii* and tobacco (*Nicotiana* sp.) plants paved the way for functional characterization of the chloroplast genes or nuclear genes encoding proteins of the photosynthetic apparatus. Using antisense RNA technology, and the resultant tobacco transgenics, with suppressed expression of the nuclear *rbc*S gene encoding the smaller subunit of Rubisco (Rodermel *et al*., 1988), Bogorad's laboratory was the first to discover that restriction in the amount of SSU protein results in the failure of the chloroplast *rbc*L gene transcription to form normal amounts of polysomes (Rodermel *et al*., 1996). This led to deciphering, for the first time, a mechanism for integrating the expression of a chloroplast gene (*rbc*L) and a nuclear gene (*rbc*S) product of a multimeric chloroplast component, that is, the Rubisco comprising LSU and SSUs. The *rbc*S antisense tobacco mutants have served to analyse the impact of varying levels of Rubisco on leaf photosynthesis and even on plant development under a range of environmental conditions (reviewed in Rodermel, 1999).

Continuing with his deep interest in interaction between nucleus and chloroplasts, Bogorad with his group investigated an interesting puzzle: Why some genes for chloroplast proteins are present in nuclear genome while other protein genes are in chloroplasts? And he sought an answer by converting a chloroplast gene, an atrazine‐resistant form of *psbA*, into a functional nuclear gene, the protein product of which was translocated into the chloroplasts (Cheung *et al*., 1988).

Bogorad's observation on the light‐induced increase of chloroplast RNA polymerase activity during the greening of leaves in etiolated maize seedlings (Apel and Bogorad, 1976), led to the initiation of several experiments analysing transcriptional control of the expression of chloroplast as well as nuclear genes encoding photosynthetic proteins (Bansal *et al*., 1992; Luan and Bogorad, 1992; Purcell *et al*., 1995; Sheen and Bogorad, 1985, 1986a,b, 1987a,b, 1988; Viret *et al*., 1994; Xu *et al*., 2001). Further characterization of different components of the chloroplast‐encoded RNA polymerase has confirmed its role in independent (as compared to the nuclear genome‐encoded RNA polymerase) transcription of the chloroplast‐encoded photosynthetic genes (Troxler *et al*., 1994).

## Research leadership

Bogorad had received numerous awards and honours and occupied prominent leadership positions of key scientific bodies such as being the President of the American Society of Plant Physiologists, of the Society for Developmental Biology and of the American Association for the Advancement of Science (Table 3).

**Table 3:** Honours and awards, and organizational competence of Lawrence Bogorad

Year	Honours and Awards, and Organizational Competence
1968–1969	President, American Society of Plant Physiology (now the American Society of Plant Biology), ASPB
1968	Fellow, American Academy of Arts and Sciences
1971	Member, National Academy of Sciences, USA
1971–1974	Member, Council and Executive Committee, American Society for Cell Biology
1974–1977	Chair, Botany Section of the National Academy of Sciences, USA
1982	Stephen Hales Prize of ASPB
1982–1984	President, Society for Developmental Biology
1985	Member, American Philosophical Society
1985	Foreign member of the Royal Danish Academy of Sciences and Letters
1986–1987	President of the American Association of Advancement of Science, AAAS
1987–1988	Member, Advisory Committee for the Biological, Behavioural, and Social Sciences, Directorate at National Science Foundation, USA
1987–1988	Member, Policy Advisory Group for the U.S. Department of Agriculture, USA
1989–1992	Member of the Council, National Academy of Sciences, USA
1900–1992	Member, Committee on Science Engineering and Public Policy, National Academies of Sciences, Engineering, and Medicine, USA
1991–1995	Editor, Proceedings of the National Academy of Sciences (PNAS)
1995	Chairman, Editorial Board of Proceedings of the National Academy of Sciences
1976	Organized the NATO (North Atlantic Treaty Organization) Workshop on the biochemistry of plant nucleic acids and proteins ‐ the first international meeting on plant molecular biology with Jacques Weil
1985	Organized the Cold Spring Harbor Symposium on ‘Molecular Biology of the Photosynthetic Apparatus’
1980	Chairman, First Gordon Conference, dedicated to Plant Molecular Biology
2004	Distinguished Service Award from the University of Chicago's Alumni Association (posthumously)

Lawrence Bogorad was elected as a member of several prominent science academies, which included the American Academy of Arts and Sciences in 1968, the National Academy of Sciences in 1971 and the American Philosophical Society in 1985. He served on the editorial board of the Proceedings of the National Academy of Sciences (PNAS), USA, first as a member, and later as chairperson of its editorial board during 1991–1995. To promote the use of molecular biology tools in advancing agriculture, Bogorad played a key role on the board of directors of the Boyce Thompson Institute and as member of the science advisory board of the Plant Genetic Systems, Belgium. He also had served on the board of Chlorogen, the first biotechnology company based on chloroplast gene expression.

## **Messages of remembrance from Dr. Bogorad**'**s Mentees (order as received)**

### Jen Sheen (sheen@molbio.mgh.harvard.edu), Professor, Department of Molecular Biology, Massachusetts General Hospital, Harvard University, Boston, USA

**Figure 7:**
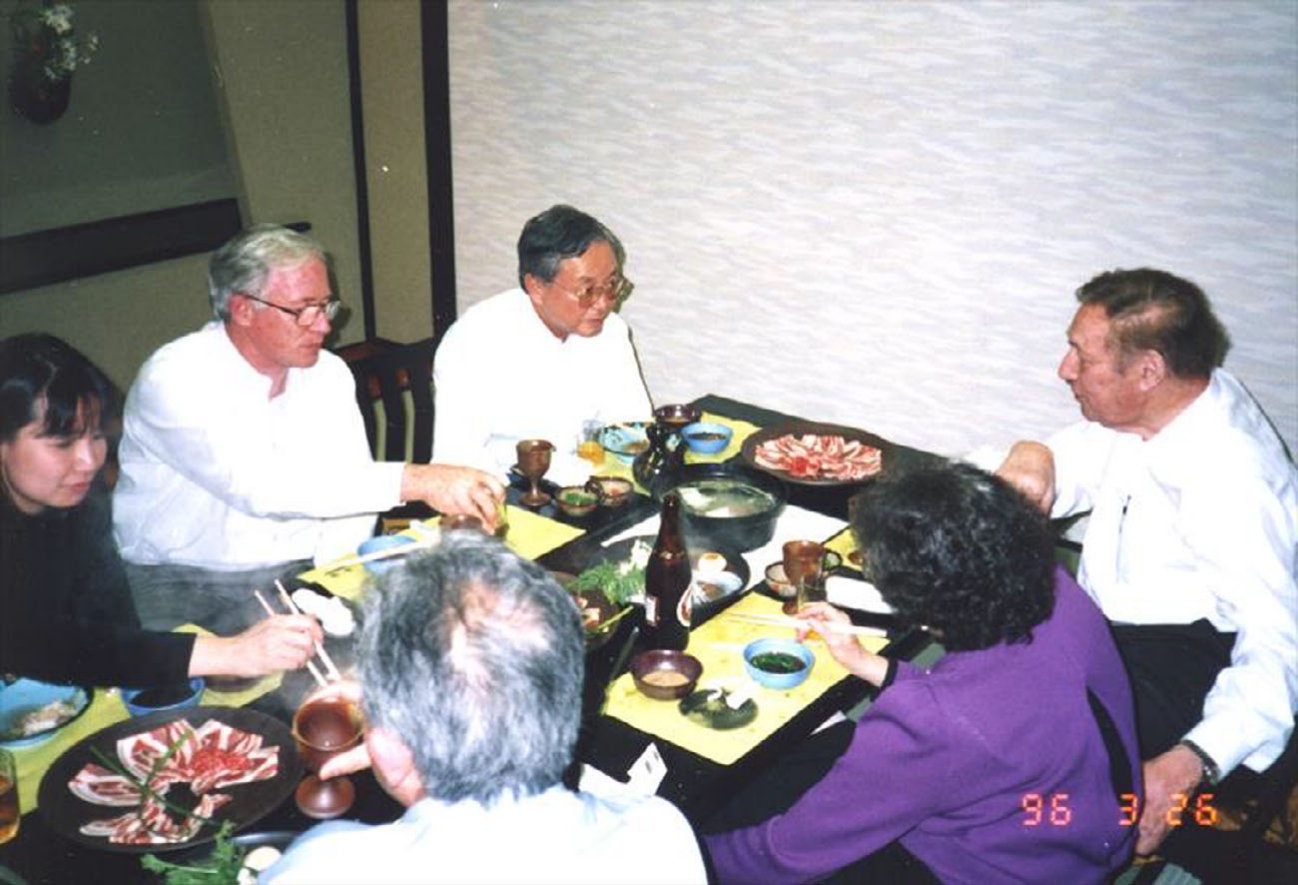
Lawrence Bogorad in discussion with (from the left to the right): Jen Sheen, Aaron Kaplan and Shigetoh Miyachi. Seen in the foreground are Teruo Ogawa and Rosalyn Bogorad, Japan, 1996 (Source: Hiro Kobayashi, Japan).

I am extremely grateful to Dr. Bogorad who created a stimulating and friendly research environment for a first‐year graduate student freshly arrived in Cambridge (MA) from Taiwan. Both Dr. Bogorad and I (see Figures 4 and 7) were fascinated by the discovery of plant C4 photosynthesis. Despite the lab focus being on the chloroplast genome in the early 1980s, I wished to explore the molecular regulation of nuclear genes in the dimorphic bundle sheath and mesophyll cells of C4 leaves. Dr. Bogorad generously provided the critical support unprecedented for a junior graduate student, which allowed me total freedom to enter an adventure in systematic cloning and identification of 15 nuclear genes involved in maize C4 photosynthesis. The research freedom also opened the door for future development of mesophyll protoplasts as a versatile and powerful plant cell‐based system to study the molecular mechanisms underlying light, organ, cell‐type‐specific, metabolic, hormonal and stress regulation of nuclear genes in maize as a C4 model plant circumventing the lack of traditional maize genetics on C4 photosynthesis.

### Néstor Carrillo (carrillo@ibr-conicet.gov.ar), Institute of Molecular and Cellular Biology of Rosario, Argentina

In 1982, I had obtained a fellowship to spend a postdoctoral period in Cambridge, United Kingdom. Then, the Falkland–Malvinas War had erupted and I had to look for another place. In time, I was accepted as a postdoc at Lawrence Bogorad's lab in Harvard, through an UNESCO fellowship. What a fortunate turn of events! There, I continued and finished research on the replication of chloroplast DNA that had been initiated by Bert Gold. By the time I joined Laurie Bogorad's lab, I had received extensive training in biochemistry and molecular biology, initially in my *alma mater*, the University of Rosario, Argentina, under the guidance of Rubén Vallejos, and during two different stays in Germany, with Wolfgang Junge at Osnabrück University and with Reinhold Herrmann at Düsseldorf University. From these three remarkable scientists, I had learnt the joy and excitement of scientific discovery and considered myself an accomplished researcher. Then, my expectations when going to Harvard were just to learn some new techniques and approaches in a world‐top lab. Man, was I wrong? My relationship with Laurie Bogorad turned out to be the most influential for my scientific career, and the time I spent at Cambridge and Boston, one of the happiest periods of my academic life. As I discovered soon, Bogorad had an amazing ability to distinguish the important from the mere interesting in a scientific result or hypothesis. In the areas of photosynthesis, genetics and molecular ecology, he was the most insightful biologist I ever met. With his kind manners and imposing voice, he taught me this all important lesson, which I have tried to follow the rest of my career, with variable success. As a bonus for me, Bogorad had attracted bright students from all over the world, and to go to his lab and mingle and learn from such brilliant people became a morning thrill. For me, it was a magical period. I am therefore really happy to have this opportunity to express my love and admiration for Lawrence Bogorad, who was *Magister Ludi* in the *Glasperlenspiel* of science.

### Hirokazu Kobayashi (hirokoba@u-shizuoka-ken.ac.jp), Professor Emeritus, University of Shizuoka, Japan

Bogorad had the greatest impact on me, the graduate student working on the regulation of expression of photosynthesis genes, to learn of his publications after 1976 which showed cloning and sequencing of pieces of maize chloroplast DNA. Near the end of my doctoral course, I applied for a fellowship for a postdoctoral study abroad newly founded by Japan Society for the Promotion of Science (JSPS). Bogorad, who was a friend of my supervisor, Takashi Akazawa, was kind enough to write an acceptable recommendation letter for me and send it to JSPS. I had a chance to work at his laboratory from 1983 to 1984 (see Figure 5), until there was a fire on 4 June 1984. The gene location was at that time named with restriction enzymes and orders of sizes generated with them, for example, ‘Bam8’ encoded PG32. Bogorad named ‘PG’ for ‘photogenes’ which were expressed in a light‐dependent manner. We now know that PG32 represents 32‐kDa protein in the photosystem II reaction centre. Along with sequencing and identifying important genes, he was interested in the regulation of their expression, and focused on the disordered expression of chloroplast genes in maize nuclear mutants in collaboration with Don Miles at the University of Missouri. Together with Enno Krebbers, I was involved in that project. I continuously inquired into the mechanism of light regulation, and then found and published, in 1977, the light‐dependent expression of nuclear genes for sigma factors of chloroplast RNA polymerase in *Arabidopsis* and, in 2010, the participation of phosphorylation and de‐phosphorylation of sigma factors in the gene‐specific transcription of photogenes in response to the redox state of plastoquinone.

### Sheng Luan (sluan@berkeley.edu), Professor and Chair, Department of Plant and Microbial Biology, University of California, Berkeley, USA

**Figure 8:**
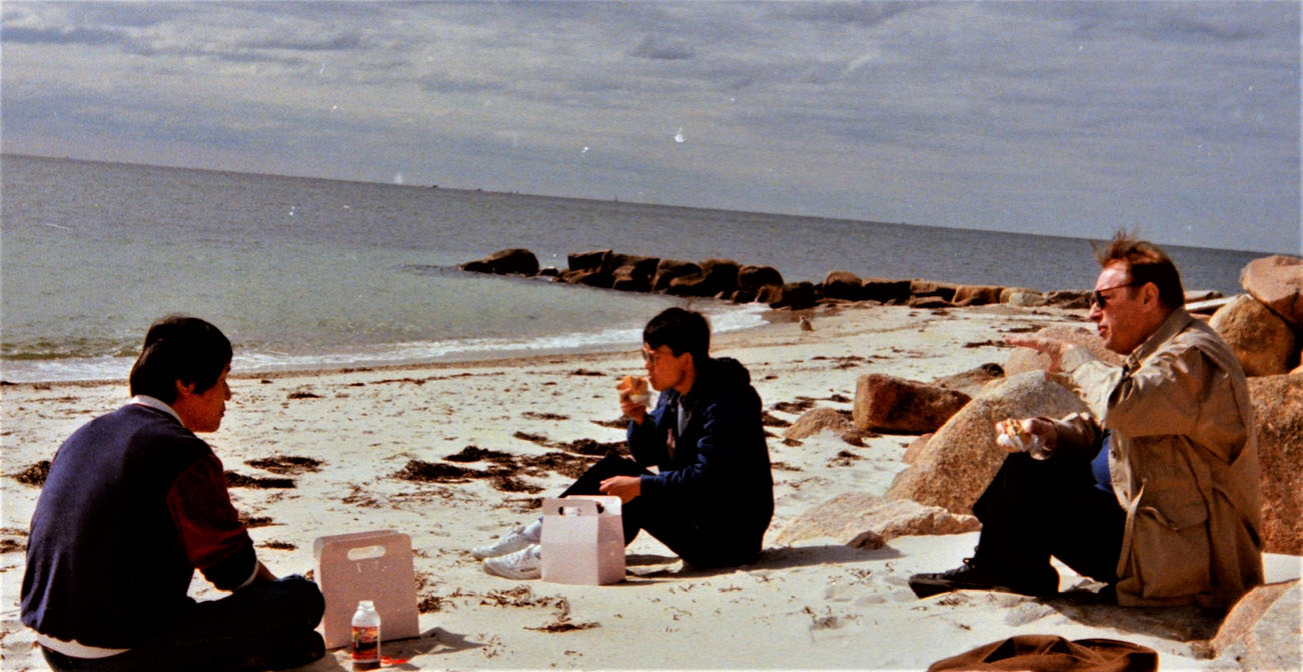
Lawrence Bogorad at the Cape Cod Beach, MA, USA, after a departmental retreat in 1990. Others seen in the picture are his Lab members, Young Park (on the left) and Sheng Luan (Source: Sheng Luan, Berkeley, CA, USA).

Starting in a Ph.D. program at Harvard Medical School, I decided to transfer to the main campus in Cambridge for my interest in plant biology. I talked with Dr. Bogorad, who happily took me into his group (see Figures 3 and 8). The Bogorad lab was the largest in the Biolab building and most of the people were senior visiting scholars and postdocs who were very well trained. I was the youngest and knew nothing about molecular biology. I began by learning about basic techniques in molecular biology and biochemistry from many people in the lab. Dr. Bogorad was then President of AAAS and travelled a lot. However, whenever I saw him around, he would talk to me about experiments and my progress. He always started with a nice smile and a warm handshake before our conversations, giving me a lot of encouragement and confidence. He spent much time correcting my thesis, teaching me about attention to the details. I learned from his example and trained my own students and postdocs in a similar way. I moved to Harvard Chemistry to do my postdoc and kept close touch with the Bogorad lab until I moved on to UC Berkeley. When I got my tenure in 1999, Dr. Bogorad wrote to congratulate me ‘13 years well spent!’ referring my first year at Harvard in 1986. Every time I passed by Boston Area, I always went back to Harvard Yard and the Biolab. I would enjoy a moment to catch some sunshine by leaning on the large rhino statues in front of the building where I had started my career.

### Gerhard Link (gerhard.link@rub.de), Emeritus Professor, Ruhr‐Universität Bochum, Germany

As a young visiting grad student with a stipend from my home university in Hannover (Germany), I had a first chance of becoming exposed to the spirit of Professor Lawrence Bogorad and his group (see Figure 5) at Harvard for thee exciting months during the winter term of 1972–1973. I well remember LB (as he was named among lab members) warmly welcoming the new student and briefly adding a generous ‘you help us and we help you’ (of course I was aware that the benefit would be fully on my side). After finishing my PhD thesis at Hannover, I then ‘returned’ to the Harvard lab as a postdoc for 3 years (1976–1979), that is, at the right time to see the onset of the pioneering phase of chloroplast molecular biology driven by the—just upcoming—powerful cloning, mapping and sequencing techniques. In retrospect, this was perhaps the most exciting period in my own scientific life, and I still gratefully remember how ingeniously Dr. Bogorad managed to bring people together for effective collaboration in challenging and highly successful projects. For me, it always was a great delight to hear his brilliant and future‐directed lectures on work from his lab at international conferences throughout many subsequent years.

### Fred Ausubel (ausubel@molbio.mgh.harvard.edu), Professor of Genetics Emeritus, Harvard Medical School, Boston, USA

Dr. Bogorad played an essential role in launching me on my academic career. Near the end of my PhD training in phage lambda genetics at MIT, I made what in those days was probably considered the curious decision to apply the tools of molecular biology to problems in agricultural science. Specifically, I set as my postdoctoral goal the transfer of functional nitrogen fixation (*nif*) genes from the prokaryotes to the plants. To achieve this goal, I needed a mentor who was willing to take the risk of letting me pursue this ambitious goal. Fortunately, Dr. Bogarad agreed to accept me as a postdoctoral fellow (see Figure 4) to pursue the *nif* gene transfer, even though the project had no direct relationship to any work that was going on in his laboratory. Moreover, he made the decision to allow me to pursue the project about a year before the discovery of recombinant DNA. After only 3 years into my postdoctoral fellowship, before the *nif* genes were cloned, Dr. Bogorad strongly advocated for me to be appointed as an Assistant Professor in the Cellular and Developmental Biology Department at Harvard. Although I thought that I was not yet sufficiently far along in my postdoctoral project to apply for faculty positions, I got the job. It was an amazing career opportunity that opened many doors for me over the years. The *nif* gene transfer project never came to fruition, but I am sure that Dr. Bogorad was not really all that interested in nitrogen fixation or its potential practical applications. He just wanted to give me a chance to show that I could make significant contributions to the field of plant molecular biology, which is what being a good mentor is all about.

### Dolores Maria Abarca (mdolores.abarca@uah.es), Universidad de Alcalá, Madrid, Spain

I spent 3 years, from 1990 to 1992, as a postdoc in the Bogorad lab at Harvard (see Figures 3 and 4). Dr. Bogorad welcomed me and my limited English, as I was launched into a multicultural marvel where you could have a thrilling discussion with scientists from all over the world over coffee, ice cream or Paula's homemade popcorn.

Lawrence Bogorad struck me as a brilliant, open‐minded scientist that created an environment where everybody was respectfully listened to and challenged. His office was always open for anything, from a silly question to a promising discovery. During my time in his lab, I could witness how new photosynthesis questions were addressed in cyanobacteria, algae and plants. The combination of different experimental systems and researchers from diverse backgrounds ranging from undergraduate students to visiting professors was the perfect breeding ground to encourage a cross‐cut approach to research. His contribution to plant knowledge goes beyond scientific discoveries, since we all left his lab with an invaluable mindset that enriches our work.

### Jeffrey N. Davidson (jndavid@uky.edu), University of Kentucky, Kentucky, USA

Dr. Bogorad had the wisdom to give three graduate students (Laurie Mets, Maureen Hanson and myself) the opportunity to expand his interests in chloroplasts to the model system *Chlamydomonas reinhardtii*. What an exciting and productive time it was for us! All three were rewarded with wonderful careers in biochemistry, genetics and molecular biology. I appreciated all his support.

### Donald Coen (don_coen@hms.harvard.edu), Harvard Medical School, Boston, USA

Although I was a graduate student in another laboratory (Alex Rich's) at a different institution (MIT), Dr. Bogorad treated me as one of his own students, and was really the main faculty mentor for my thesis research. He was very kind—and also very funny!

### Richard Sayre (rsayre@newmexicoconsortium.org), New Mexico Consortium, Los Alamos, USA

On the occasion of the 100^th^ year since Dr. Bogorad's birth, it is good to recall his impact on myself and others. I joined the Bogorad lab in the fall of 1982 and left in 1986. This was the first large lab environment I had experienced. There were over 30 postdocs, graduate students and technicians in the group from all corners of the globe (see Figure 5). They were exciting times. Not the least of which was the lab fire. Nearly half the lab was lost in the fire, and many students lost all their work. But it was also a time when we all pulled together both during the inventory and clean‐up as well as rebuilding a new lab. Ever the eternal optimist, Dr. Bogorad was initially devastated by the loss, but soon he was leading us to a new home in the building and cheering us all on to do our best. Within 3 months, we were fully operational. There remain three indelible impressions I have of his personal style and science. First, we were all one big family. Dr. Bogorad truly cared not only about our work as individuals and team members but our families as well. He also knew each of the more than 30 projects inside out. But the major lasting impression I was left with was his inquisitiveness, sense of wonder and his ability to reinvent his science to always stay at the forefront. The Bogorad family of students and associates remains most grateful for those experiences.

### Andre Steinmetz (asteinme@pt.lu), LIH Luxembourg Institute of Health, CRP Santé, Luxembourg

I am delighted that I have this opportunity to express my gratitude to Dr. Bogorad on his 100^th^ birthday anniversary. He made excellent contributions to the science of photosynthesis, as a pioneering scientist. Dr. Bogorad has been quite helpful in my career development and I found him a very caring person (Figure 5). I always appreciated the guidance received from him. I still have fond memories of my years in his lab, which were definitely the best and most exciting in my scientific career.

### Jean Viret (jeanviret@yahoo.com), Blade Therapeutics, Inc., South San Francisco, USA

Dr. Bogorad was a true humanist. I keep from him (a least) three values. Everyone is absolutely equal; he was welcoming, every day, and could truly connect with anyone, which made the lab very collaborative and productive (see Figures 3 and 4). Make your writing absolutely clear; as an editor of many journals, he taught us how to write better scientific articles. Learn from others and through your interactions with others; he continued to learn from all his collaborators and would thank them for it.

### Joseph Seckbach (joseph.seckbach@mail.huji.ac.il), Graduated University of Chicago under LB in 1965, Israel

Although over 55 years have passed since I graduated the University of Chicago and received my MSc and PhD in the Department of Botany, under the guidance of Professor Lawrence Bogorad (LB), it seems to me that I finished the University just yesterday. LB welcomed me when I entered the gate of the Department of Botany, during my first days in the United States, to arrange my programme of weekly graduate courses. When we first met, he was 40 years old and had been at U of C for 8 years already.

We became quite friendly (although I respected him and always addressed him by his formal academic title; and not Laurie as others called him, using his nickname).

I was also close to the Bogorad family. When our department had a gathering at his home, his wife prepared kosher refreshments for me since she knew that I kept dietary laws. When LB came to give a series of lectures at the Hebrew University in Jerusalem, I met his wife Rosalyn and his mother and drove them around the city, including a visit to the Western Wall.

After LB moved to Harvard University, I did my best to visit him several times and he hosted me in his home. He waited and welcomed me in the train station and drove me to Lexington, Massachusetts, where he lived.

In the late 1960, I was working at UCLA, under Professor Willard. F. Libby (Nobel Laureate 1960) in the Geophysical Department. My target was to find conditions in which microorganisms could thrive in similar life as it was assumed then on the planet Venus. I was looking for many microorganisms that could tolerate some conditions that matched Venus, such as CO_2_ atmosphere, warm media and under pressure. Many microorganisms could not take such extremophilic conditions. I approached several scientists, but only LB advised me to try his ‘favourite’ red alga *Cyanidium caldarium* for my project, and cells, from this alga, were really perfect for our project: They survived in acidic media, high temperatures, thrived in pure CO_2_ atmosphere as well as under pressure. Following this study, several articles were published, some with the head of the unit, Professor Libby, and others with my colleagues. This project at UCLA moved me into Space research, into extremophilic microorganisms and to Astrobiology to this day. So, it was LB who assisted me in the UCLA project and led to my editing numerous volumes with co‐editors on possibilities for life on other planets and working on Astrobiology.

In the volume of *Diatoms Fundamentals and Applications* (2019) edited by J. Seckbach & R. Gordon, published by Wiley‐Scrivener, I have published another dedication to LB (see pages v‐vi).

### Olivier Vallon (ovallon@ibpc.fr), Institut de Biologie Physico‐Chimique CNRS, Paris, France

‘Larry’ we called him in his absence, but it was ‘Dr. Bogorad’ to him. A towering figure, physically impressive, but always smiling and courteous. To me, a young postdoc arriving from Europe in 1987, with zero knowledge of molecular biology, he offered an opportunity to explore new horizons. If I made no big discovery while in his lab, it was not his fault. I came with precise ideas on what I wanted to do; not so good it seems in retrospect. He encouraged me to pursue them, gave me access to a flock of rabbits and peptides to make antibodies from. He introduced me to Dan Branton, another great scientist of the Harvard Biolabs, who let me use his electron microscope facilities. As was his custom with the postdocs, Dr. Bogorad gave me full freedom, and a wonderful environment to work in. The lab was recovering from the ‘Big Fire’ which had destroyed a lot of the work of my fellow lab mates, Valdis Dzelzkalns, Steve Rodermel and others. Still, the atmosphere was not of gloom, but of rebirth. Dr. Bogorad's early life, of which he did not speak much but which we knew had been full of danger, of rivers crossed and hurdles vanquished, was an inspiration: Science was just an adventure among others.

### Uwe Klein (uwe.klein@ibv.uio.no), University of Oslo, Oslo, Norway

I came to Dr. Bogorad's lab at Harvard University in Cambridge in the fall of 1989 (see Figures 3 and 5). At that time, it became evident that molecular biology methods would contribute significantly to progress in life sciences. Being formerly educated as a plant physiologist, I spent more than 3 years of learning and experimenting with various molecular biology techniques under Dr. Bogorad's guidance. He gave me a lot of freedom in choosing research directions. I also remember fondly all the people I met in the lab, at the Biological Laboratories, and in the Cambridge area. It has been a memorable time.

### Dr. Amita Pal (amita@jcbose.ac.in), Bose Institute, Kolkata, India

I visited Prof. Lawrence Bogorad's lab for 4 months as FAO visiting fellow under the UNDP project to transfer foreign gene in mung bean using the particle gun in the year 1990. The Biolistic™ PDS‐1000 Particle Delivery System assembled by M/s DuPont was available in Prof. Bogorad's Department at Harvard University, USA. My own interest was to work there to gain insights in Plant Biology through his brilliant mentorship as well as from the learned lab mates. Prior to my visit to the Harvard University, Prof. Bogorad visited Bose Institute, India, to evaluate our UNDP project. At that time I had a chance to interact with him and I found him very cordial, affectionate and amicable in nature. While discussing about the problems, we were then encountering with Agrobacterium‐mediated gene transfer in mung bean, he himself invited me to work in his lab. I spent a wonderful time under his mentorship. He was very caring and affectionate. Although he was very busy with his academic activities, yet he took personal care of my well‐being during my stay. I am ever grateful to him for his proficient mentorship and consider the short visit, to his lab, as one of the best academic ventures in my academic life.

### Alice Cheung (acheung@umass.edu), Professor, Department of Biochemistry and Molecular Biology, University of Massachusetts, Amherst, USA

A quote from a previously written remembrance (Eulogy of Lawrence Bogorad, January 2, 2004, Harvard University, Cambridge, MA, USA)

I think I was most affected by Dr. Bogorad's immense optimism, the energy and devotion he committed to the advancement of science, his love in the learning of different topics and all things good and fun ‐‐ even down to the reports of our little day to day progress and small parties in the lab, the trust and support he provided for his students and associates, and unyielding loyalty even years after they have left the lab.

I would not forget how, at good times, Dr. Bogorad told us we should enjoy our results and push forward with all the possibilities, until and unless future efforts told us we were on the wrong track. Nor would I forget how, at bad times and we tended to complain, he told us people were the most adaptable and we should learn to work with and do the best with what we've got. His curiosity would always lead him to urge us to ‘just give it a try, there is little to lose in trying’. These were apparently how he had conducted his life himself, as he talked about his career choices in his 2001 Plant Physiology article ‘Samples from Fifty years of Career Decisions’. I learned to love the exploration; the price sometimes is high, but the journey of learning makes it all worthwhile.

In the mid 80's, we were a group of almost 20 postdocs and several graduate students. His ability and, probably even more so his attentiveness, to provide everyone's project a fair share of his input, created a surprisingly collegial atmosphere when fierce competition among us could have prevailed. Even what some would observe to be slight partiality that existed was cleverly exploited positively by all of us in the lab to advance our common good, i.e. to get what we wanted. And Dr. Bogorad would go along with our little games and bought us the new or fancy toys that we might not really have needed.

The mid 80's was a period of great excitement in learning. It was a period of emerging possibilities in the manipulation of plant genomes, with each of us immersed in pursuing new frontiers in scientific adventures and in our careers. But it was also one with devastation when the lab caught fire one morning. At its aftermath, it was Dr. Bogorad's optimism that carried the day. Despite the tremendous difficulty he himself was facing to rebuild the lab, Dr. Bogorad still was the biggest cheer leader for us all. And the lab emerged stronger, especially in the ties it forged among those of us who had experienced through it together.

As the years go by, I found myself more and more admiring of Dr. Bogorad's unceasing love of adventure, not just in science but also in life. It was amazing to observe how his positive outlook in life never seemed to be tainted by cynicism from life's experience.

With his death, I continued to be inspired by the consistency Dr. Bogorad had lived his life. The eulogies given by his family and the fond memories that so many have sent to his family to mourn the loss have all pointed to a life of passionate dedication to what were important to him, family, friends, associates and colleagues, a fiercely competitive work ethics for his own career advancement and the advancement of science, yet embodied in a most modest humanity that always took time and effort to attend to others. I will miss him dearly’.

### James D. DeCamp (jddecamp@clarkelbing.com), Partner, CLARK+ELBING LLP, Boston, USA

Sometime in the late spring of 1989, I first met Prof. Bogorad in his Bio Labs office overlooking the greenhouses off Divinity Ave (see Figures 3 and 4). After a quick hello, he put down his dictation microphone and jumped out from behind his desk. ‘The gene gun is on the 3rd floor, let's go!’ he said excitedly. He led me in an elevator down to a small room on the third floor. On a table was gun 002, a squarish machine of metal and plastic connected to a vacuum pump, made by Cornell's John Sanford. Powered by a 22‐caliber nail gun cartridge, the gun delivered its DNA‐coated tungsten payload into all sorts of targets growing in the Bogorad lab. As he carefully cleaned the machine, he made sure to tell me, with a smile, that I could buy the cartridges at Roach's Sporting Goods (and gun shop) on Mass Ave in Porter Sq. Not wasting a second, we were then off to meet a very friendly Alan Blowers, who would for the next few days tutor me on his project and gun etiquette.

I returned in September (of 1989) for a 3‐year stay, working with all the worldwide actors assembled by Prof. Bogorad. His enthusiasm was ever contagious, as was his warmth and hospitality. I am grateful for that time in the Bio Labs. I remember him saying on more than one occasion that he wished he would be around for the next phase in biology. One can only wonder what contributions he would be making today!

### Kailash C. Bansal (kcbansal27@gmail.com), National Academy of Agricultural Sciences, New Delhi, India

Having worked in Dr. Bogorad's lab was, for me, a dream come true. My stay in those years was most productive and proved extremely helpful in my career advancement.

After completing my Ph.D. in Plant Physiology from the Indian Agricultural Research Institute, New Delhi, in 1989, my dream was to go for a postdoc in a good lab for learning the basics and pursuing advanced research in plant molecular biology. Fortunately, I got an opportunity to work in Dr. Bogorad's lab at Harvard Biolabs during the period 1990–1992 (see Figures 3 and 4). I joined the group working on understanding the molecular regulation of nuclear photosynthetic gene expression in mesophyll versus bundle sheath cells in maize leaves. Bogorad's group was establishing a system using protoplasts but I wanted to do it differently using the whole maize leaf, and talked to him about it. He immediately agreed and gave me freedom to work independently, and even arranged a big microtome for leaf sectioning to detect the expression of the reporter gene in the two cell types. We were finally successful in establishing the whole leaf assay with a clear distinction in expression driven by mesophyll versus bundle sheath cell‐specific promoters in the respective cells. I discovered that Dr. Bogorad was a passionate teacher and a great mentor.

About his caring nature: As I could not afford a car, Dr. Bogorad would invariably drop me at my home in Arlington, and importantly, wait for my work for the day to be over only to drop me home. This was amazing and I used to enjoy these rides and conversations with him. He would come to my bench around 8.30 pm and ask me—‘KC, are you ready to go home?’, and if I had work still to do, I would politely ask him to go ahead as I have work still to finish. But to my pleasant surprise, sometime later I will find him still sitting in his office room, obviously waiting for me. Then, I will go to him after completing the work and say, ‘Yes Dr. Bogorad, I am ready to go home’. Despite being a well‐renowned scientist with seminal contributions, and awards and honours to his credit, I found Dr. Bogorad to be a real nice human being with a very helpful and caring attitude.

How privileged I was to be associated with such a legendary scientist and a gentle soul! I was able to spend some quality time with Dr. Bogorad in January 2003 when he so kindly agreed to my request and visited New Delhi for delivering a plenary talk in an International Plant Physiology Congress (see Figure 2). That was my wish fulfilled and remains as solace for me and my family meeting him after about a decade and just less than a year before he breathed his last.

### Henry Daniell (hdaniell@upenn.edu), W.D. Miller Professor, Vice Chair, School of Dental Medicine, University of Pennsylvania, Philadelphia, USA

On his 100^th^ birthday, I express my deep gratitude and appreciation to Laurie as a great role model in both professional and personal life. My first interaction with him started with a phone call from him when I was at Washington State University, after my research on foreign gene expression in chloroplasts was published in PNAS (Daniell and McFadden, 1987). I was shocked when he asked me why I did not respond to his invitation. I misunderstood that his letter was a formal appreciation of my publication but did not realize that he really invited me to Harvard. I was truly humbled when he came to Boston airport to pick me up, despite flight delays. After paying for several months for my stay at the Radcliff guesthouse, he communicated the research I performed in his lab to PNAS (Daniell *et al*., 1990), but declined authorship stating that this would be helpful in advancing my career and that I had pioneered this concept. Until this day, I truly regret missing the opportunity to be his co‐author, especially because this research was done in his lab and with his intellectual input. But he fulfilled my wishes by visiting every one of the universities I worked in (see Figure 6), encouraging my students ‘to run with it, even if they had one good day in the year’ or served on the board of company I found or participated in workshops that I organized, including those abroad that involved very long international flights. He set a very high bar for mentorship and it is quite challenging to emulate his selfless guidance. He believed so much in advancing chloroplast transformation that he cited five among 10 references on this topic in the introductory chapter of his book on the Molecular Biology of Plastids (Bogorad and Vasil, 1991). Although foreign genes were first integrated into the Chlamydomonas chloroplast genome, delay in achieving foreign protein accumulation in Chlamydomonas chloroplasts stimulated his interest in advancing foreign protein expression in plant chloroplasts. I hope on his 100^th^ birthday he will be proud of advances made in this field that he had pioneered (Daniell *et al*., 2021). Beyond his extraordinary scientific accomplishments, I am truly thankful for educating me to be a good mentor.

### Satish C. Maheshwari^#^ and Sudhir K. Sopory (sopory@icgeb.res.in), SERB (Govt. of India) Distinguished Fellow, ICGEB, New Delhi, India ^#^Deceased; see Pareek *et al*. (2020)

A quote from Current Science (Bogorad, 2001b):

In this world there are very few who are able to leave their footprints on the sands of time. Lawrence Bogorad is one of them – brilliant and charismatic, well known for his dedication to science and commitment to excellence. He exudes warmth and kindness, and his magnanimous, winsome personality has always attracted a continuous stream of students to work in the congenial atmosphere of his lab at Harvard University, where he has spent the most productive years of his life….

**Figure 9:**
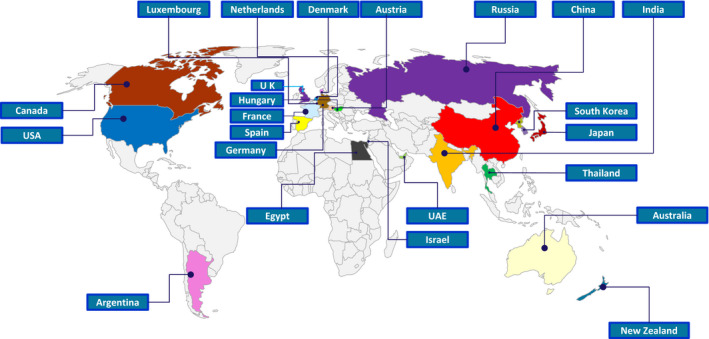
Mapping the countries around the world from where Professor Lawrence Bogorad's Lab attracted graduate students, postdoctoral fellows, visiting scholars and scientists.

Lawrence Bogorad (1921–2003) is fondly remembered for his groundbreaking contributions in science and for mentoring hundreds of graduate students, postdoctoral fellows and visiting scholars & scientists drawn from around the world; he has made an indelible mark in the international community (Figures 7 and 9). We remember him fondly, and with great respect, on his 100^th^ birth anniversary.

## Conflict of interest

Author declares no conflict of interest.

## References

[pbi13719-bib-0001] Apel, K. and Bogorad, L. (1976) Light‐induced increase in the activity of maize plastid DNA‐dependent RNA polymerase. Eur. J. Biochem. 67, 615–620. 964260 10.1111/j.1432-1033.1976.tb10727.x

[pbi13719-bib-0002] Bansal, K.C. and Bogorad, L. (1993) Cell type‐preferred expression of maize cab‐m1: repression in bundle sheath cells and enhancement in mesophyll cells. Proc. Natl Acad. Sci. USA, 90, 4057–4061. 8483921 10.1073/pnas.90.9.4057 PMC46445

[pbi13719-bib-0003] Bansal, K.C. , Viret, J.‐F. , Haley, J. , Khan, B.M. , Schantz, R. and Bogorad, L. (1992) Transient expression from cab‐m1 and rbcS‐m3 promoter sequences is different in mesophyll and bundle sheath cells in maize leaves. Proc. Natl Acad. Sci. USA, 89, 3654–3658. 1565662 10.1073/pnas.89.8.3654 PMC48927

[pbi13719-bib-0004] Bedbrook, J.R. and Bogorad, L. (1976) Endonuclease recognition sites mapped on Zea mays chloroplast DNA. Proc. Natl Acad. Sci. USA, 73, 4309–4313. 16592373 10.1073/pnas.73.12.4309 PMC431441

[pbi13719-bib-0005] Bedbrook, J.R. , Coen, D.M. , Beaton, A.R. , Bogorad, L. and Rich, A. (1979) Location of the single gene for the large subunit of ribulose bisphosphate carboxylase on the maize chloroplast chromosome. J. Biol. Chem. 254, 905–910. 762099

[pbi13719-bib-0006] Bedbrook, J.R. , Link, G. , Coen, D.M. , Bogorad, L. and Rich, A. (1978) Maize plastid gene expressed during photoregulated development. Proc. Natl Acad. Sci. USA, 75, 3060–3064. 16592541 10.1073/pnas.75.7.3060 PMC392713

[pbi13719-bib-0007] Bedbrook, R. , Kolodner, R. and Bogorad, L. (1977) Zea mays chloroplast ribosomal RNA genes are part of a 22,000 base pair inverted repeat. Cell, 11, 739–749. 890739 10.1016/0092-8674(77)90288-4

[pbi13719-bib-0008] Blowers, A.D. , Bogorad, L. , Shark, K.B. and Sanford, J.C. (1989) Studies on *Chlamydomonas* chloroplast transformation: foreign DNA can be stably maintained in the chromosome. Plant Cell, 1, 123. 2535460 10.1105/tpc.1.1.123 PMC159743

[pbi13719-bib-0009] Blowers, A.D. , Ellmore, G.S. , Klein, U. and Bogorad, L. (1990) Transcriptional analysis of endogenous and foreign genes in chloroplast transformants of Chlamydomonas. Plant Cell, 2, 1059. 2152108 10.1105/tpc.2.11.1059 PMC159954

[pbi13719-bib-0010] Blowers, A.D. , Klein, U. , Ellmore, G.S. and Bogorad, L. (1993) Functional in vivo analyses of the 3’ flanking sequences of the Chlamydomonas chloroplast rbcL and psaB genes. Mol. Gen. Genet. 238, 339–349. 8388079 10.1007/BF00291992

[pbi13719-bib-0011] Bogorad, L. (1958a) The enzymatic synthesis of porphyrins from porphobilinogen. I. Uroporphyrin I. J. Biol. Chem. 233, 501–509. 13563528

[pbi13719-bib-0012] Bogorad, L. (1958b) The enzymatic synthesis of porphyrins from porphobilinogen. II. Uroporphyrin III. J. Biol. Chem. 233, 510–515. 13563529

[pbi13719-bib-0013] Bogorad, L. (1960) The biosynthesis of protochlorophyll. In Comparative Biochemistry of Photoreactive Systems ( Allen, M.B. , ed.), pp. 226–256. New York, NY: Academic Press Inc.

[pbi13719-bib-0014] Bogorad, L. (1975) Evolution of organelles and eukaryotic genomes. Science, 188, 891. 1138359 10.1126/science.1138359

[pbi13719-bib-0078] Bogorad, L. (1982) Regulation of intracellular gene flow in the evolution of eukaryotic genomes. In On the Origins of Chloroplasts ( Schiff, J.A. , ed.), pp. 277–295. New York, NY: Elsevier/North‐Holland, Inc.

[pbi13719-bib-0015] Bogorad, L. (2000) Engineering chloroplasts: an alternative site for foreign genes, proteins, reactions and products. Trends Biotechnol. 18, 257–263. 10802561 10.1016/s0167-7799(00)01444-x

[pbi13719-bib-0016] Bogorad, L. (2001a) Emergence of plant molecular biology viewed through the portal of chloroplast research. Curr. Sci. 80, 153.

[pbi13719-bib-0017] Bogorad, L. (2001b) Samples from fifty years of career decisions. Plant Physiol. 126, 1345–1346. 11500533 10.1104/pp.126.4.1345 PMC1540129

[pbi13719-bib-0018] Bogorad, L. (2003). Photosynthesis research: advances through molecular biology – the beginnings, 1975–1980’s and on…. Photosynth. Res. 76, 13. 16228563 10.1023/A:1024957602990

[pbi13719-bib-0019] Bogorad, L. (2005) Photosynthesis research: advances through molecular biology‐ the beginnings, 1975–1980s and on. In Discoveries in Photosynthesis ( Govindjee , Beatty, J.T. , Gest, H. and Allen, J.F. , eds.), pp. 1025–1045. Dordrecht. The Netherlands: Springer.

[pbi13719-bib-0020] Bogorad, L. and Granick, S. (1952) Precursors of protoporphyrin in chlorella. Nature, 170, 321. 12993148 10.1038/170321a0

[pbi13719-bib-0021] Bogorad, L. and Granick, S. (1953) The enzymatic synthesis of porphyrins from porphobilinogen. Proc. Natl Acad. Sci. USA, 39, 1176. 16589396 10.1073/pnas.39.12.1176 PMC1063934

[pbi13719-bib-0022] Bogorad, L. and Marks, G.S. (1960a) Studies on the biosynthesis of uroporphyrin III from porphobilinogen and the behaviour of uroporphyrin esters in paper chromatography. Biochim. Biophys. Acta, 41, 356. 13802156 10.1016/0006-3002(60)90024-x

[pbi13719-bib-0023] Bogorad, L. and Marks, G.S. (1960b) The enzymatic synthesis of uroporphyrins from porphobilinogen. IV. Investigations on the participation of formaldehyde. J. Biol. Chem. 235, 2127–2129. 13802157

[pbi13719-bib-0024] Bogorad, L. and Vasil, I.K. (1991) The Molecular Biology of Plastids. San Diego, CA: Academic Press.

[pbi13719-bib-0025] Boynton, J.E. , Gillham, N.W. , Harris, E.H. , Hosier, J.P. , Johnson, A.M. , Jones, A.R. , Randolph‐Anderson, B.L. *et al*. (1988) Chloroplast transformation in *Chlamydomonas* with high velocity microprojectiles. Science, 240, 1534. 2897716 10.1126/science.2897716

[pbi13719-bib-0026] Carpenter, A.T. and Scott, J.J. (1959) The relationship of opsopyrroledicarboxylic acid to the biosynthesis of porphyrin. Biochem. J. 71, 325. 13628573 10.1042/bj0710325 PMC1196793

[pbi13719-bib-0027] Cheung, A.Y. , Bogorad, L. , Van Montagu, M. and Schell, J. (1988) Relocating a gene for herbicide tolerance: a chloroplast gene is converted into a nuclear gene. Proc. Natl Acad. Sci. USA, 85, 391–395. 16593905 10.1073/pnas.85.2.391 PMC279554

[pbi13719-bib-0028] Coen, D.M. , Bedbrook, J.R. , Bogorad, L. and Rich, A. (1977) Maize chloroplast DNA fragment encoding the large subunit of ribulosebisphosphate carboxylase. Proc. Natl Acad. Sci. USA, 74, 5487–5491. 16592473 10.1073/pnas.74.12.5487 PMC431774

[pbi13719-bib-0029] Daniell, H. , Jin, S. , Zhu, X.‐G. , Gitzendanner, M.A. , Soltis, D.E. and Soltis, P.S. (2021) Green giant – a tiny chloroplast genome with mighty power to produce high‐value proteins: history and phylogeny. Plant Biotechnol. J. 19, 430–447. 33484606 10.1111/pbi.13556 PMC7955891

[pbi13719-bib-0030] Daniell, H. and McFadden, B.A. (1987) Uptake and expression of bacterial and cyanobacterial genes by isolated cucumber etioplasts. Proc. Natl Acad. Sci. USA, 84, 6349–6353. 3114748 10.1073/pnas.84.18.6349 PMC299073

[pbi13719-bib-0031] Daniell, H. , Vivekananda, J. , Nielsen, B.L. , Ye, G.N. , Tewari, K.K. and Sanford, J.C. (1990) Transient foreign gene expression in chloroplasts of cultured tobacco cells after biolistic delivery of chloroplast vectors. Proc. Natl Acad. Sci. USA, 87, 88–92. 2404285 10.1073/pnas.87.1.88 PMC53205

[pbi13719-bib-0032] Davidson, J.N. , Hanson, M.R. and Bogorad, L. (1974) An altered chloroplast ribosomal protein in ery‐M1 mutants of *Chlamydomonas reinhardi* . Mol. Gen. Genet. 132, 119. 4421915 10.1007/BF00272177

[pbi13719-bib-0033] Fish, L.E. and Bogorad, L. (1986) Identification and analysis of the maize P700 chlorophyll a apoproteins PS I‐A1 and PS I‐A2 by high pressure liquid chromatography. Analysis and partial sequence determination. J. Biol. Chem. 261, 8134–8139. 3522564

[pbi13719-bib-0034] Fish, L.E. , Kuck, U. and Bogorad, L. (1985) Two partially homologous adjacent light‐inducible maize chloroplast genes encoding polypeptides of the P700 chlorophyll a‐protein complex of Photosystem I. J. Biol. Chem. 260, 1413–1421. 3881431

[pbi13719-bib-0035] Haley, J. and Bogorad, L. (1989) A 4‐kDa maize chloroplast polypeptide associated with the cytochrome b6‐f complex: subunit 5, encoded by the chloroplast petE gene. Proc. Natl Acad. Sci. USA, 86, 1534–1538. 2922397 10.1073/pnas.86.5.1534 PMC286732

[pbi13719-bib-0036] Hanson, M.R. and Bogorad, L. (1977a) Effects of erythromycin on membrane‐bound chloroplast ribosomes from wild type *Chlamydomonas reinhardi* and erythromycin‐resistant mutants. Biochim. Biophys. Acta, 479, 279–289. 921999 10.1016/0005-2787(77)90110-1

[pbi13719-bib-0037] Hanson, M.R. and Bogorad, L. (1977b) Complementation analysis at the ery‐m1 locus in *Chlamydomonas reinhardi* . Mol. Gen. Genet. 153, 271–277.

[pbi13719-bib-0038] Hanson, M.R. and Bogorad, L. (1978) The *ery*‐M2 group of *Chlamydomonas reinhardii*: cold‐sensitive, erythromycin‐resistant mutants deficient in chloroplast ribosomes. J. Gen. Microbiol. 105, 253.

[pbi13719-bib-0039] Hu, J. and Bogorad, L. (1990) Maize chloroplast RNA polymerase: the 180‐, 120‐, and 38‐kilodalton polypeptides are encoded in chloroplast genes. Proc. Natl Acad. Sci. USA, 87, 1531–1535. 2304916 10.1073/pnas.87.4.1531 PMC53509

[pbi13719-bib-0040] Hu, J. , Troxler, R.F. and Bogorad, L. (1991) Maize chloroplast RNA polymerase: the 78‐kilodalton polypeptide is encoded by the plastid rpoC1 gene. Nucleic Acids Res. 12, 3431. 10.1093/nar/19.12.3431 PMC328344 2062657

[pbi13719-bib-0041] Jacobson, A.B. , Swift, H. and Bogorad, L. (1963) Cytochemical studies concerning the occurrence and distribution of RNA in plastids of *Zea mays* . J. Cell Biol. 17, 557. 17533669 10.1083/jcb.17.3.557 PMC2106211

[pbi13719-bib-0042] Jordan, P.M. and Shemin, D. (1973) Purification and properties of uroporphyrinogen I synthetase from *Rhodopseudomonas spheroids* . J. Biol. Chem. 248, 1019. 4539746

[pbi13719-bib-0043] Klein, U. , De Camp, J.D. and Bogorad, L. (1992) Two types of chloroplast gene promoters in *Chlamydomonas reinhardtii* . Proc. Natl Acad. Sci. USA, 89, 3453–3457. 1565638 10.1073/pnas.89.8.3453 PMC48886

[pbi13719-bib-0044] Klein, U. , Salvador, M.L. and Bogorad, L. (1994) Activity of the Chlamydomonas chloroplast rbcL gene promoter is enhanced by a remote sequence element. Proc. Natl Acad. Sci. USA, 91, 10819–10823. 7971968 10.1073/pnas.91.23.10819 PMC45117

[pbi13719-bib-0045] Krebbers, E. , Larrinua, I.M. , McIntosh, L. and Bogorad, L. (1982) The maize chloroplast genes for the b and e subunits of the photosynthetic coupling factor CF1 are fused. Nucleic Acids Res. 10, 4985. 6290998 10.1093/nar/10.16.4985 PMC320846

[pbi13719-bib-0046] Link, G. , Coen, D.M. and Bogorad, L. (1978) Differential expression of the gene for the large subunit of ribulose bisphosphate carboxylase in maize leaf cell types. Cell, 15, 725–731. 728987 10.1016/0092-8674(78)90258-1

[pbi13719-bib-0047] Luan, S. and Bogorad, L. (1992) A rice cab gene promoter contains separate cis‐acting elements that regulate expression in dicot and monocot plants. Plant Cell, 8, 971. 10.1105/tpc.4.8.971 PMC160189 1392604

[pbi13719-bib-0048] McIntosh, L. , Poulsen, C. and Bogorad, L. (1980) Chloroplast gene sequence for the large subunit of ribulose bisphosphate carboxylase of maize. Nature, 288, 556–560.

[pbi13719-bib-0049] Merchant, S. (2009) Lawrence Bogorad, 1921–2003, A Biographical Memoir. Washington, DC: National Academy of Sciences.

[pbi13719-bib-0050] Merchant, S. and Bogorad, L. (1986a) Regulation by copper of the expression of plastocyanin and cytochrome c552 in *Chlamydomonas reinhardi* . Mol. Cell. Biol. 6, 462–469. 3023849 10.1128/mcb.6.2.462-469.1986 PMC367534

[pbi13719-bib-0051] Merchant, S. and Bogorad, L. (1986b) Rapid degradation of apoplastocyanin in Cu(II)‐deficient cells of *Chlamydomonas reinhardtii* . J. Biol. Chem. 261, 15850–15853. 3023330

[pbi13719-bib-0052] Merchant, S. and Bogorad, L. (1987) Metal ion regulated gene expression: use of a plastocyanin‐less mutant of *Chlamydomonas reinhardtii* to study the Cu(II)‐dependent expression of cytochrome c‐552. EMBO J. 6, 2531–2535. 2824187 10.1002/j.1460-2075.1987.tb02540.x PMC553670

[pbi13719-bib-0053] Mets, L.J. and Bogorad, L. (1971) Mendelian and uniparental alterations in erythromycin binding by plastid ribosomes. Science, 174, 707–709. 5123420 10.1126/science.174.4010.707

[pbi13719-bib-0054] Mets, L.J. and Bogorad, L. (1972) Altered chloroplast ribosomal proteins associated with erythromycin‐resistant mutants in two genetic systems of *Chlamydomonas reinhardtii* . Proc. Natl Acad. Sci. USA, 69, 3779. 4509340 10.1073/pnas.69.12.3779 PMC389872

[pbi13719-bib-0055] Nichols, K.E. and Bogorad, L. (1960) Studies on phycobilin formation with mutants of *Cyanidium caldarium* . Nature, 188, 870–872. 10.1038/188870b0 13728770

[pbi13719-bib-0056] Pareek, A. , Soni, V. , Sopory, S.K. , Khurana, J.P. , Sowjanya Sree, K. , Tyagi, A.K. , Narsimhan, S. *et al*. (2020) Satish Chandra Maheshwari (1933–2019) – a brilliant, passionate and an outstanding shining light for all of plant biology. Physiol. Mol. Biol. Plants, 26, 1087–1098. 32549674 10.1007/s12298-020-00794-2 PMC7266906

[pbi13719-bib-0057] Purcell, M. , Mabrouk, Y.M. and Bogorad, L. (1995) Red/far‐red and blue light‐responsive regions of maize rbcS‐m3 are active in bundle sheath and mesophyll cells, respectively. Proc. Natl Acad. Sci. USA, 92, 11504–11508. 8524792 10.1073/pnas.92.25.11504 PMC40430

[pbi13719-bib-0058] Rodermel, S. (1999) Subunit control of Rubisco biosynthesis – a relic of an endosymbiont past? Photosynth. Res. 59, 105.

[pbi13719-bib-0059] Rodermel, S.R. , Abbott, M.S. and Bogorad, L. (1988) Nuclear organelle interactions: nuclear antisense gene inhibits ribulose bisphosphate carboxylase enzyme levels in transformed tobacco plants. Cell, 55, 673–681. 3052855 10.1016/0092-8674(88)90226-7

[pbi13719-bib-0060] Rodermel, S.R. and Bogorad, L. (1985) Maize plastid photogenes: mapping and photoregulation of transcript levels during light‐induced development. J. Cell Biol. 100, 463–476. 2981888 10.1083/jcb.100.2.463 PMC2113432

[pbi13719-bib-0061] Rodermel, S.R. , Haley, J. , Jiang, C.J. , Tsai, C.H. and Bogorad, L. (1996) A mechanism for intergenomic integration: abundance of ribulose bisphosphate carboxylase small‐subunit protein influences the translation of the large‐subunit mRNA. Proc. Natl Acad. Sci. USA, 93, 3881. 8632983 10.1073/pnas.93.9.3881 PMC39453

[pbi13719-bib-0062] Rodermel, S.R. , Viret, J.‐F. and Krebbers, E. (2005) Lawrence Bogorad (1921–2003), a pioneer in photosynthesis research: a tribute. Photosynth. Res. 83, 17. 16143903 10.1007/s11120-004-6316-5

[pbi13719-bib-0063] Salvador, M.L. , Klein, U. and Bogorad, L. (1993) 5’ sequences are important positive and negative determinants of the longevity of Chlamydomonas chloroplast gene transcripts. Proc. Natl Acad. Sci. USA, 90, 1556–1560. 8434017 10.1073/pnas.90.4.1556 PMC45913

[pbi13719-bib-0064] Sheen, J.‐Y. and Bogorad, L. (1985) Differential expression of the ribulose bisphosphate carboxylase large subunit gene in bundle sheath and mesophyll cells of developing maize leaves is influenced by light. Plant Physiol. 79, 1072–1076. 16664532 10.1104/pp.79.4.1072 PMC1075029

[pbi13719-bib-0065] Sheen, J.‐Y. and Bogorad, L. (1986a) Expression of ribulose bisphosphate carboxylase large subunit and three small subunit genes in two cell types of maize leaves. EMBO J. 13, 3417. 10.1002/j.1460-2075.1986.tb04663.x PMC1167374 16453739

[pbi13719-bib-0066] Sheen, J.‐Y. and Bogorad, L. (1986b) Differential expression of six light‐harvesting chlorophyll a/b binding protein genes in maize leaf cell types. Proc. Natl Acad. Sci. USA, 83, 7811–7815. 3532122 10.1073/pnas.83.20.7811 PMC386812

[pbi13719-bib-0067] Sheen, J.‐Y. and Bogorad, L. (1987a) Regulation of levels of nuclear transcripts for C4 photosynthesis in bundle sheath and mesophyll cells of maize leaves. Plant Mol. Biol. 8, 227–238. 24301127 10.1007/BF00015031

[pbi13719-bib-0068] Sheen, J.‐Y. and Bogorad, L. (1987b) Differential expression of C4 pathway genes in mesophyll and bundle sheath cells of greening maize leaves. J. Biol. Chem. 262, 11726–11730. 2442151

[pbi13719-bib-0069] Sheen, J.‐Y. and Bogorad, L. (1988) Differential expression in bundle sheath and mesophyll cells of maize of genes for photosystem II components encoded by the plastid genome. Plant Physiol. 86, 1020. 16666025 10.1104/pp.86.4.1020 PMC1054621

[pbi13719-bib-0070] Sheen, J.‐Y. , Sayre, R. and Bogorad, L. (1987) Differential expression of oxygen‐evolving polypeptide genes in maize leaf cell types. Plant Mol. Biol. 9, 217. 24276970 10.1007/BF00166458

[pbi13719-bib-0071] Singh, M. , Boutanev, A. , Zucchi, P. and Bogorad, L. (2001) Gene elements that affect the longevity of rbcL sequence‐containing transcripts in *Chlamydomonas reinhardtii* chloroplasts. Proc. Natl Acad. Sci. USA, 98, 2289–2294. 11226232 10.1073/pnas.041609798 PMC30131

[pbi13719-bib-0072] Steinmetz, A. , Gubbins, E.J. and Bogorad, L. (1982) The anticodon of the maize chloroplast gene for tRNALeu is split by a large intron. Nucleic Acids Res. 10, 3027. 6285285 10.1093/nar/10.10.3027 PMC320685

[pbi13719-bib-0073] Subramanian, A.R. , Steinmetz, A. and Bogorad, L. (1983) Maize chloroplast DNA encodes a protein sequence homologous to the bacterial ribosome assembly protein S4. Nucleic Acids Res. 11, 5277–5286. 6308577 10.1093/nar/11.15.5277 PMC326263

[pbi13719-bib-0074] Swift, H. (1985) Lawrence Bogorad: president‐elect of the AAAS. Science, 229, 353–354. 17795885 10.1126/science.229.4711.353

[pbi13719-bib-0075] Troxler, R.F. , Zhang, F. , Hu, J. and Bogorad, L. (1994) Evidence that sigma factors are components of chloroplast RNA polymerase. Plant Physiol. 104, 753–759. 8159791 10.1104/pp.104.2.753 PMC159255

[pbi13719-bib-0076] Viret, J.‐F. , Mbrouk, Y. and Bogorad, L. (1994) Transcriptional photoregulation of cell‐type‐preferred expression of maize rbcS‐m3: 3¢ and 5¢ sequences are involved. Proc. Natl Acad. Sci. USA, 91, 8577–8581. 8078926 10.1073/pnas.91.18.8577 PMC44649

[pbi13719-bib-0077] Xu, T. , Purcell, M. , Zucchi, P. , Helentjaris, T. and Bogorad, L. (2001) TRM1, a YY1‐like suppressor of rbcS‐m3 expression in maize mesophyll cells. Proc. Natl Acad. Sci. USA, 98, 2295–2300. 11226233 10.1073/pnas.041610098 PMC30132

